# (Formato-κ*O*)bis­(1,10-phenanthroline-κ^2^ *N*,*N*′)copper(II) formate hexa­hydrate

**DOI:** 10.1107/S1600536808035320

**Published:** 2008-11-08

**Authors:** Wei Xu, Jian-Li Lin, Hong-Zhen Xie, Ming Zhang

**Affiliations:** a State Key Laboratory Base of Novel Functional Materials & Preparation Science, Faculty of Materials Science and Chemical Engineering, Ningbo University, Ningbo 315211, People’s Republic of China

## Abstract

In the title compound, [Cu(CHO_2_)(C_12_H_8_N_2_)_2_]CHO_2_·6H_2_O, the Cu atom is coordinated in a distorted trigonal-bipyramidal fashion by an O atom of the formate ligand and four N atoms of two phenanthroline ligands with Cu—O and Cu—N distances of 2.020 (3) and 1.978 (3)–2.177 (3) Å, respectively. Hydrogen bonding O—H⋯O between water molecules and between water anions as well as π–π inter­actions [centroid–centroid distances between phen rings = 3.38 (7) and 3.40 (5) Å] are responsible for the supra­molecular assembly.

## Related literature

For backgorund on the utilization of formic acid for the rational design and synthesis of coordination polymers and the potential applications of these compounds, see: Dybtsev *et al.* (2003 ▶); Manson *et al.* (2003 ▶); Wang *et al.* (2005 ▶, 2006 ▶).
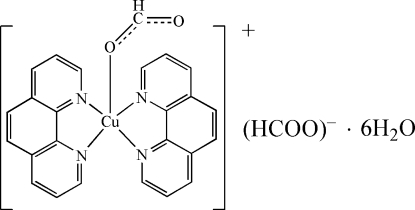


## Experimental

### 

#### Crystal data

[Cu(CHO_2_)(C_12_H_8_N_2_)_2_]CHO_2_·6H_2_O
*M* *_r_* = 622.09
Monoclinic, 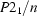

*a* = 14.765 (3) Å
*b* = 12.764 (3) Å
*c* = 15.513 (3) Å
β = 109.76 (3)°
*V* = 2751.4 (11) Å^3^
*Z* = 4
Mo *K*α radiation
μ = 0.86 mm^−1^
*T* = 295 (2) K
0.43 × 0.29 × 0.22 mm

#### Data collection

Bruker P4 diffractometer
Absorption correction: ψ scan (*XSCANS*; Siemens, 1996 ▶) *T*_min_ = 0.740, *T*_max_ = 0.819
5942 measured reflections
4812 independent reflections
3341 reflections with *I* > 2σ(*I*)
*R*_int_ = 0.068
3 standard reflections every 97 reflections intensity decay: none

#### Refinement

*R*[*F* ^2^ > 2σ(*F* ^2^)] = 0.053
*wR*(*F* ^2^) = 0.163
*S* = 1.11
4812 reflections
372 parameters
H-atom parameters constrained
Δρ_max_ = 0.67 e Å^−3^
Δρ_min_ = −0.76 e Å^−3^

### 

Data collection: *XSCANS* (Siemens, 1996 ▶); cell refinement: *XSCANS*; data reduction: *XSCANS*; program(s) used to solve structure: *SHELXS97* (Sheldrick, 2008 ▶); program(s) used to refine structure: *SHELXL97* (Sheldrick, 2008 ▶); molecular graphics: *SHELXTL* (Sheldrick, 2008 ▶); software used to prepare material for publication: *SHELXL97*.

## Supplementary Material

Crystal structure: contains datablocks global, I. DOI: 10.1107/S1600536808035320/pk2125sup1.cif

Structure factors: contains datablocks I. DOI: 10.1107/S1600536808035320/pk2125Isup2.hkl

Additional supplementary materials:  crystallographic information; 3D view; checkCIF report

## Figures and Tables

**Table 1:** Hydrogen-bond geometry (Å, °)

*D*—H⋯*A*	*D*—H	H⋯*A*	*D*⋯*A*	*D*—H⋯*A*
O5—H5*A*⋯O8	0.82	2.10	2.874 (5)	160
O5—H5*B*⋯O4^i^	0.73	2.10	2.808 (6)	164
O6—H6*A*⋯O3	0.74	2.16	2.870 (5)	163
O6—H6*B*⋯O10	0.85	2.03	2.810 (5)	153
O7—H7*A*⋯O4	0.90	1.95	2.799 (5)	158
O7—H7*B*⋯O6^ii^	0.73	2.08	2.794 (6)	165
O8—H8*A*⋯O3	0.82	2.12	2.879 (5)	154
O8—H8*B*⋯O7^i^	0.76	2.20	2.876 (6)	148
O9—H9*A*⋯O2^iii^	0.75	2.05	2.754 (5)	157
O9—H9*B*⋯O10^iv^	0.83	2.09	2.827 (6)	148
O10—H10*A*⋯O5	0.85	2.03	2.798 (6)	149
O10—H10*B*⋯O9	0.82	2.01	2.832 (6)	179

Symmetry codes: (i) 
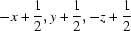
; (ii) 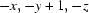; (iii) 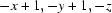; (iv) 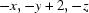.

**Fig. 1.**
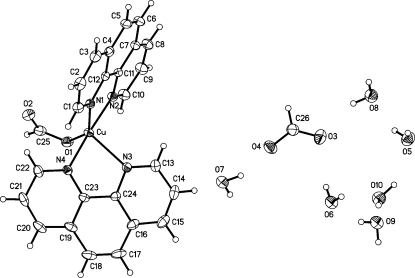
The molecular structure of the title complex showing 40% probability displacement ellipsoids.

**Fig. 2.**
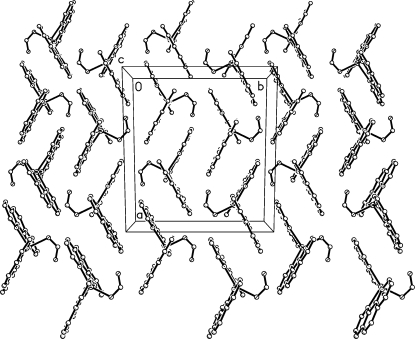
Supramolecular assembly of the [Cu(phen)2(HCOO)]+ complex cations based on π-π stacking interactions.

**Fig. 3.**
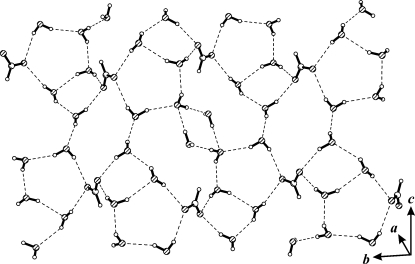
The two-dimensional water-formate anion layers.

## supplementary crystallographic information

### Comment

In recent years, interest in the utilization of formic acid for the rational design and synthesis of coordination polymers has been growing rapidly due to their potential applications and intriguing architectures (Dybtsev, *et al.*, 2003; Manson, *et al.*, 2003; Wang, *et al.*, 2005; Wang, *et al.*, 2006). In the present contribution, we report a new copper complex, [Cu(phen)_2_(HCOO)](HCOO).6H_2_O, resulting from self-assembly of Cu^2+^ ions, phenanthroline and formic acid.

The asymmetric unit of the title compound consists of one [Cu(phen)_2_(HCOO)]^+^ complex cation, one formate anion and six water molecules. As illustrated in Fig. 1, the Cu atom is penta-coordinated by four N atoms of two different bidentate chelating phen ligands and one O atom of the formate ligand. The coordination polyhedra is a trigonal bipyramid with d(Cu—O) = 2.020 (3) Å and d(Cu—N) = 1.978 (3)–2.177 (3) Å. The phenanthroline ring systems are each nearly planar and the dihedral angle between the two phen planes is 56.69 (5)°. The complex cations are arranged in such a way that non-symmetry related phen planes of neighboring complexes are oriented parallel to each other with phen-to-phen separations of about 3.38 (7) and 3.40 (5) Å. Such π-π stacking interactions assemble the complex cations into two-dimensional layers parallel to (001) (Fig. 2). The six crystallographically distinct H_2_O molecules and the non-coordinating formate anions are held together by hydrogen bonds (*d*(O···O) = 2.794 (6)–2.879 (5) Å; <O—H···O = 148–179°) to generate two-dimensional water-anionic layers parallel to (100) (Fig. 3). Through the hydrogen bonding interactions (O9···O2), the [Cu(phen)_2_(HCOO)]^+^ complex cationic layers are assembled into a three-dimensional network with the H_2_O molecules.

### Experimental

Addition of 2.0 ml (1.0 *M*) NaOH to a stirred aqueous solution of 0.171 g (1.00 mmol) CuCl_2_.2H_2_O in 5.0 ml H_2_O gave a blue precipitate, which was then separated by centrifugation, followed by washing with double-distilled water until no detectable Cl^-^ anions were present in the supernatant. The precipitate was added to a stirred ethanolic aqueous solution of 0.398 g (2.00 mmol) phenanthroline monohydrate in 20 ml EtOH/H_2_O (*v*/*v* = 1:1). To the mixture was added 2.0 ml (1.0 *M*) HCOOH and the blue suspension was further stirred for *ca* 1 h. After filtration, the filtrate (pH = 5.56) was allowed to stand at room temperature. Slow evaporation for several days gave blue block crystals (yield 32%, based on the initial CuCl_2_.2H_2_O input).

### Refinement

H atoms attached to C atoms of the phen ligands and formate anions were positioned geometrically and refined using a riding model, with C—H = 0.93, and *U*_iso_(H) values set at 1.2 *U*eq(C).  The hydrogen atoms of the water molecules were located in difference Fourier maps and placed at fixed positions with  U_iso_(H) values set at 1.2 Ueq(O).

### Crystal data

**Table:**

[Cu(CHO_2_)(C_12_H_8_N_2_)_2_]CHO_2_·6H_2_O	*F*_000_ = 1292
*M**_r_* = 622.09	*D*_x_ = 1.502 Mg m^−^^3^
Monoclinic, *P*2_1_/*n*	Mo *K*α radiation λ = 0.71073 Å
Hall symbol: -P 2yn	Cell parameters from 25 reflections
*a* = 14.765 (3) Å	θ = 5.0–12.5º
*b* = 12.764 (3) Å	µ = 0.86 mm^−^^1^
*c* = 15.513 (3) Å	*T* = 295 (2) K
β = 109.76 (3)º	Block, blue
*V* = 2751.4 (11) Å^3^	0.43 × 0.29 × 0.22 mm
*Z* = 4	

### Data collection

**Table:**

Bruker P4 diffractometer	*R*_int_ = 0.068
Radiation source: fine-focus sealed tube	θ_max_ = 25.0º
Monochromator: graphite	θ_min_ = 1.7º
*T* = 295(2) K	*h* = −1→17
θ/2θ scans	*k* = −1→15
Absorption correction: ψ scan(XSCANS; Siemens, 1996)	*l* = −18→17
*T*_min_ = 0.740, *T*_max_ = 0.819	3 standard reflections
5942 measured reflections	every 97 reflections
4812 independent reflections	intensity decay: none
3341 reflections with *I* > 2σ(*I*)	

### Refinement

**Table:**

Refinement on *F*^2^	Hydrogen site location: inferred from neighbouring sites
Least-squares matrix: full	H-atom parameters constrained
*R*[*F*^2^ > 2σ(*F*^2^)] = 0.053	*w* = 1/[σ^2^(*F*_o_^2^) + (0.0836*P*)^2^ + 2.1346*P*] where *P* = (*F*_o_^2^ + 2*F*_c_^2^)/3
*wR*(*F*^2^) = 0.163	(Δ/σ)_max_ < 0.001
*S* = 1.11	Δρ_max_ = 0.67 e Å^−^^3^
4812 reflections	Δρ_min_ = −0.76 e Å^−^^3^
372 parameters	Extinction correction: SHELXL97 (Sheldrick, 2008), Fc^*^=kFc[1+0.001xFc^2^λ^3^/sin(2θ)]^-1/4^
Primary atom site location: structure-invariant direct methods	Extinction coefficient: 0.0091 (10)
Secondary atom site location: difference Fourier map	

### Special details

**Table:**

Geometry. All e.s.d.'s (except the e.s.d. in the dihedral angle between two l.s. planes) are estimated using the full covariance matrix. The cell e.s.d.'s are taken into account individually in the estimation of e.s.d.'s in distances, angles and torsion angles; correlations between e.s.d.'s in cell parameters are only used when they are defined by crystal symmetry. An approximate (isotropic) treatment of cell e.s.d.'s is used for estimating e.s.d.'s involving l.s. planes.
Refinement. Refinement of *F*^2^ against ALL reflections. The weighted *R*-factor *wR* and goodness of fit *S* are based on *F*^2^, conventional *R*-factors *R* are based on *F*, with *F* set to zero for negative *F*^2^. The threshold expression of *F*^2^ > σ(*F*^2^) is used only for calculating *R*-factors(gt) *etc*. and is not relevant to the choice of reflections for refinement. *R*-factors based on *F*^2^ are statistically about twice as large as those based on *F*, and *R*- factors based on ALL data will be even larger.

### Fractional atomic coordinates and isotropic or equivalent isotropic displacement parameters (Å^2^)

**Table:**

	*x*	*y*	*z*	*U*_iso_*/*U*_eq_	
Cu	0.64373 (3)	0.25126 (4)	0.01020 (3)	0.0379 (2)	
N1	0.7686 (2)	0.3308 (2)	0.0792 (2)	0.0375 (7)	
N2	0.6376 (2)	0.2407 (2)	0.1353 (2)	0.0396 (7)	
C1	0.8348 (3)	0.3739 (3)	0.0496 (3)	0.0478 (10)	
H1A	0.8252	0.3736	−0.0129	0.057*	
C2	0.9184 (3)	0.4195 (3)	0.1096 (3)	0.0563 (11)	
H2A	0.9643	0.4471	0.0871	0.068*	
C3	0.9326 (3)	0.4234 (3)	0.2005 (3)	0.0558 (11)	
H3A	0.9881	0.4540	0.2405	0.067*	
C4	0.8635 (3)	0.3813 (3)	0.2342 (3)	0.0436 (9)	
C5	0.8705 (4)	0.3821 (4)	0.3286 (3)	0.0580 (12)	
H5C	0.9246	0.4112	0.3719	0.070*	
C6	0.8005 (3)	0.3416 (4)	0.3557 (3)	0.0558 (11)	
H6C	0.8056	0.3460	0.4171	0.067*	
C7	0.7191 (3)	0.2924 (3)	0.2925 (2)	0.0447 (9)	
C8	0.6439 (4)	0.2461 (4)	0.3157 (3)	0.0568 (12)	
H8C	0.6446	0.2481	0.3758	0.068*	
C9	0.5702 (4)	0.1984 (4)	0.2498 (3)	0.0592 (12)	
H9C	0.5209	0.1667	0.2650	0.071*	
C10	0.5685 (3)	0.1968 (4)	0.1599 (3)	0.0531 (10)	
H10C	0.5174	0.1642	0.1155	0.064*	
C11	0.7114 (3)	0.2880 (3)	0.2003 (2)	0.0359 (8)	
C12	0.7831 (3)	0.3341 (3)	0.1702 (2)	0.0357 (8)	
N3	0.5138 (2)	0.3447 (2)	−0.0467 (2)	0.0414 (7)	
N4	0.6447 (2)	0.2693 (2)	−0.1166 (2)	0.0393 (7)	
C13	0.4500 (3)	0.3792 (3)	−0.0117 (3)	0.0532 (10)	
H13A	0.4635	0.3737	0.0512	0.064*	
C14	0.3627 (3)	0.4240 (4)	−0.0651 (4)	0.0647 (13)	
H14A	0.3186	0.4462	−0.0381	0.078*	
C15	0.3430 (4)	0.4348 (3)	−0.1563 (4)	0.0661 (13)	
H15A	0.2851	0.4648	−0.1922	0.079*	
C16	0.4094 (3)	0.4007 (3)	−0.1966 (3)	0.0526 (11)	
C17	0.3954 (4)	0.4059 (4)	−0.2923 (3)	0.0679 (15)	
H17A	0.3393	0.4362	−0.3316	0.081*	
C18	0.4601 (4)	0.3687 (4)	−0.3271 (3)	0.0656 (14)	
H18A	0.4480	0.3730	−0.3898	0.079*	
C19	0.5478 (3)	0.3223 (3)	−0.2694 (3)	0.0514 (11)	
C20	0.6173 (4)	0.2797 (4)	−0.3019 (3)	0.0608 (13)	
H20A	0.6090	0.2827	−0.3640	0.073*	
C21	0.6962 (4)	0.2345 (3)	−0.2430 (3)	0.0596 (13)	
H21A	0.7426	0.2059	−0.2643	0.072*	
C22	0.7083 (4)	0.2306 (3)	−0.1504 (3)	0.0520 (11)	
H22A	0.7635	0.1994	−0.1106	0.062*	
C23	0.5649 (3)	0.3155 (3)	−0.1749 (2)	0.0392 (9)	
C24	0.4947 (3)	0.3552 (3)	−0.1378 (2)	0.0384 (8)	
C25	0.6441 (4)	0.0437 (3)	−0.0195 (3)	0.0552 (11)	
H25	0.6237	−0.0242	−0.0378	0.066*	
O1	0.5806 (2)	0.1097 (2)	−0.02386 (19)	0.0543 (7)	
O2	0.7306 (2)	0.0600 (3)	0.0067 (2)	0.0683 (9)	
C26	0.1997 (4)	0.5352 (4)	0.2316 (3)	0.0618 (12)	
H26	0.2453	0.5124	0.2859	0.074*	
O3	0.1314 (3)	0.5838 (3)	0.2400 (3)	0.0798 (10)	
O4	0.2164 (3)	0.5125 (3)	0.1609 (2)	0.0796 (11)	
O5	0.1030 (3)	0.9175 (3)	0.3028 (3)	0.0833 (11)	
O6	0.0247 (3)	0.6818 (3)	0.0693 (2)	0.0770 (10)	
O7	0.1697 (3)	0.3535 (3)	0.0289 (2)	0.0729 (10)	
O8	0.1647 (3)	0.7213 (3)	0.3948 (2)	0.0719 (9)	
O9	0.1259 (3)	1.0518 (3)	0.0279 (2)	0.0885 (12)	
O10	0.0479 (3)	0.8961 (3)	0.1125 (3)	0.0831 (11)	
H5A	0.1169	0.8562	0.3155	0.100*	
H5B	0.1448	0.9521	0.3137	0.100*	
H6A	0.0482	0.6457	0.1074	0.100*	
H6B	0.0514	0.7399	0.0896	0.100*	
H7A	0.1700	0.4114	0.0616	0.100*	
H7B	0.1178	0.3552	0.0052	0.100*	
H8A	0.1746	0.6809	0.3576	0.100*	
H8B	0.2154	0.7354	0.4264	0.100*	
H9A	0.1669	1.0365	0.0126	0.100*	
H9B	0.0905	1.0691	−0.0241	0.100*	
H10A	0.0478	0.9198	0.1640	0.100*	
H10B	0.0707	0.9418	0.0885	0.100*	

### Atomic displacement parameters (Å^2^)

**Table:**

	*U*^11^	*U*^22^	*U*^33^	*U*^12^	*U*^13^	*U*^23^
Cu	0.0408 (3)	0.0447 (3)	0.0282 (3)	−0.0022 (2)	0.0116 (2)	0.00049 (18)
N1	0.0403 (17)	0.0364 (16)	0.0372 (16)	0.0011 (14)	0.0149 (13)	0.0014 (13)
N2	0.0380 (17)	0.0473 (18)	0.0350 (16)	−0.0030 (14)	0.0141 (13)	0.0018 (13)
C1	0.054 (2)	0.045 (2)	0.049 (2)	−0.0029 (19)	0.0223 (19)	0.0063 (18)
C2	0.048 (2)	0.050 (2)	0.076 (3)	−0.005 (2)	0.028 (2)	0.004 (2)
C3	0.043 (2)	0.047 (2)	0.072 (3)	−0.0049 (19)	0.010 (2)	−0.008 (2)
C4	0.041 (2)	0.036 (2)	0.048 (2)	0.0021 (17)	0.0074 (18)	−0.0051 (17)
C5	0.065 (3)	0.054 (3)	0.040 (2)	0.007 (2)	−0.003 (2)	−0.0094 (19)
C6	0.070 (3)	0.060 (3)	0.032 (2)	0.006 (2)	0.011 (2)	−0.0045 (19)
C7	0.057 (2)	0.046 (2)	0.0314 (19)	0.0104 (19)	0.0154 (18)	0.0028 (17)
C8	0.071 (3)	0.069 (3)	0.040 (2)	0.007 (2)	0.032 (2)	0.009 (2)
C9	0.067 (3)	0.069 (3)	0.052 (3)	−0.008 (2)	0.034 (2)	0.010 (2)
C10	0.050 (2)	0.064 (3)	0.048 (2)	−0.010 (2)	0.021 (2)	0.001 (2)
C11	0.039 (2)	0.0367 (18)	0.0308 (18)	0.0066 (16)	0.0103 (16)	0.0029 (15)
C12	0.039 (2)	0.0308 (17)	0.0362 (18)	0.0061 (16)	0.0116 (16)	0.0012 (15)
N3	0.0456 (18)	0.0380 (17)	0.0403 (17)	0.0032 (14)	0.0142 (15)	0.0006 (13)
N4	0.0449 (18)	0.0409 (17)	0.0354 (16)	−0.0010 (14)	0.0181 (14)	0.0006 (13)
C13	0.058 (3)	0.047 (2)	0.060 (3)	0.002 (2)	0.027 (2)	−0.005 (2)
C14	0.054 (3)	0.051 (3)	0.095 (4)	0.007 (2)	0.033 (3)	−0.010 (3)
C15	0.055 (3)	0.041 (2)	0.091 (4)	0.006 (2)	0.009 (3)	0.007 (2)
C16	0.051 (2)	0.033 (2)	0.061 (3)	−0.0014 (18)	0.003 (2)	0.0078 (19)
C17	0.080 (4)	0.045 (3)	0.051 (3)	−0.003 (2)	−0.012 (3)	0.018 (2)
C18	0.091 (4)	0.056 (3)	0.035 (2)	−0.015 (3)	0.002 (2)	0.009 (2)
C19	0.079 (3)	0.041 (2)	0.0320 (19)	−0.018 (2)	0.015 (2)	−0.0006 (17)
C20	0.103 (4)	0.051 (2)	0.036 (2)	−0.023 (3)	0.033 (3)	−0.0063 (19)
C21	0.094 (4)	0.047 (2)	0.059 (3)	−0.007 (2)	0.053 (3)	−0.009 (2)
C22	0.068 (3)	0.047 (2)	0.052 (2)	−0.001 (2)	0.036 (2)	−0.0015 (19)
C23	0.052 (2)	0.0323 (19)	0.0311 (18)	−0.0075 (17)	0.0105 (17)	−0.0003 (15)
C24	0.041 (2)	0.0306 (18)	0.0382 (19)	−0.0027 (15)	0.0070 (16)	0.0032 (15)
C25	0.075 (3)	0.037 (2)	0.042 (2)	−0.004 (2)	0.006 (2)	0.0013 (18)
O1	0.0549 (17)	0.0500 (17)	0.0495 (16)	−0.0009 (15)	0.0064 (13)	0.0002 (13)
O2	0.061 (2)	0.076 (2)	0.0609 (19)	0.0112 (18)	0.0103 (16)	0.0038 (17)
C26	0.074 (3)	0.047 (2)	0.058 (3)	−0.002 (2)	0.015 (2)	−0.002 (2)
O3	0.065 (2)	0.074 (2)	0.103 (3)	0.0053 (19)	0.031 (2)	−0.007 (2)
O4	0.116 (3)	0.060 (2)	0.061 (2)	0.001 (2)	0.027 (2)	−0.0082 (16)
O5	0.089 (3)	0.075 (2)	0.090 (3)	0.017 (2)	0.036 (2)	0.018 (2)
O6	0.078 (2)	0.086 (3)	0.064 (2)	0.004 (2)	0.0206 (18)	−0.0112 (19)
O7	0.080 (2)	0.076 (2)	0.0580 (19)	0.0064 (19)	0.0182 (17)	−0.0100 (17)
O8	0.075 (2)	0.075 (2)	0.072 (2)	−0.0008 (18)	0.0341 (19)	0.0044 (18)
O9	0.085 (3)	0.108 (3)	0.067 (2)	0.016 (2)	0.0197 (19)	−0.012 (2)
O10	0.094 (3)	0.075 (2)	0.076 (2)	0.009 (2)	0.023 (2)	0.0016 (19)

### Geometric parameters (Å, °)

**Table:**

Cu—N2	1.978 (3)	C14—H14A	0.9300
Cu—N4	1.986 (3)	C15—C16	1.400 (7)
Cu—O1	2.020 (3)	C15—H15A	0.9300
Cu—N1	2.059 (3)	C16—C24	1.407 (5)
Cu—N3	2.177 (3)	C16—C17	1.430 (7)
N1—C1	1.332 (5)	C17—C18	1.333 (7)
N1—C12	1.356 (5)	C17—H17A	0.9300
N2—C10	1.327 (5)	C18—C19	1.430 (7)
N2—C11	1.352 (5)	C18—H18A	0.9300
C1—C2	1.397 (6)	C19—C20	1.398 (7)
C1—H1A	0.9300	C19—C23	1.403 (5)
C2—C3	1.355 (6)	C20—C21	1.343 (7)
C2—H2A	0.9300	C20—H20A	0.9300
C3—C4	1.402 (6)	C21—C22	1.388 (6)
C3—H3A	0.9300	C21—H21A	0.9300
C4—C12	1.399 (5)	C22—H22A	0.9300
C4—C5	1.432 (6)	C23—C24	1.437 (5)
C5—C6	1.345 (6)	C25—O2	1.220 (5)
C5—H5C	0.9300	C25—O1	1.245 (5)
C6—C7	1.415 (6)	C25—H25	0.9300
C6—H6C	0.9300	C26—O3	1.228 (6)
C7—C11	1.396 (5)	C26—O4	1.237 (6)
C7—C8	1.409 (6)	C26—H26	0.9300
C8—C9	1.360 (7)	O5—H5A	0.8162
C8—H8C	0.9300	O5—H5B	0.7309
C9—C10	1.386 (6)	O6—H6A	0.7368
C9—H9C	0.9300	O6—H6B	0.8486
C10—H10C	0.9300	O7—H7A	0.8961
C11—C12	1.421 (5)	O7—H7B	0.7303
N3—C13	1.311 (5)	O8—H8A	0.8225
N3—C24	1.352 (5)	O8—H8B	0.7656
N4—C22	1.316 (5)	O9—H9A	0.7471
N4—C23	1.353 (5)	O9—H9B	0.8279
C13—C14	1.397 (6)	O10—H10A	0.8544
C13—H13A	0.9300	O10—H10B	0.8217
C14—C15	1.351 (7)		
			
N2—Cu—N4	176.56 (12)	C24—N3—Cu	108.7 (2)
N2—Cu—O1	91.46 (12)	C22—N4—C23	118.4 (3)
N4—Cu—O1	90.09 (12)	C22—N4—Cu	126.6 (3)
N2—Cu—N1	81.60 (12)	C23—N4—Cu	114.6 (2)
N4—Cu—N1	98.78 (12)	N3—C13—C14	122.7 (4)
O1—Cu—N1	146.07 (12)	N3—C13—H13A	118.7
N2—Cu—N3	96.24 (12)	C14—C13—H13A	118.7
N4—Cu—N3	80.52 (12)	C15—C14—C13	119.4 (4)
O1—Cu—N3	96.83 (12)	C15—C14—H14A	120.3
N1—Cu—N3	116.87 (12)	C13—C14—H14A	120.3
C1—N1—C12	118.0 (3)	C14—C15—C16	120.1 (4)
C1—N1—Cu	131.1 (3)	C14—C15—H15A	120.0
C12—N1—Cu	110.8 (2)	C16—C15—H15A	120.0
C10—N2—C11	118.6 (3)	C15—C16—C24	116.6 (4)
C10—N2—Cu	127.2 (3)	C15—C16—C17	125.0 (4)
C11—N2—Cu	114.1 (2)	C24—C16—C17	118.4 (4)
N1—C1—C2	121.9 (4)	C18—C17—C16	122.1 (4)
N1—C1—H1A	119.0	C18—C17—H17A	118.9
C2—C1—H1A	119.0	C16—C17—H17A	118.9
C3—C2—C1	120.0 (4)	C17—C18—C19	121.0 (4)
C3—C2—H2A	120.0	C17—C18—H18A	119.5
C1—C2—H2A	120.0	C19—C18—H18A	119.5
C2—C3—C4	119.8 (4)	C20—C19—C23	117.3 (4)
C2—C3—H3A	120.1	C20—C19—C18	123.7 (4)
C4—C3—H3A	120.1	C23—C19—C18	119.0 (4)
C12—C4—C3	116.8 (4)	C21—C20—C19	119.7 (4)
C12—C4—C5	118.7 (4)	C21—C20—H20A	120.2
C3—C4—C5	124.6 (4)	C19—C20—H20A	120.2
C6—C5—C4	121.3 (4)	C20—C21—C22	119.9 (4)
C6—C5—H5C	119.3	C20—C21—H21A	120.1
C4—C5—H5C	119.3	C22—C21—H21A	120.1
C5—C6—C7	121.0 (4)	N4—C22—C21	122.6 (5)
C5—C6—H6C	119.5	N4—C22—H22A	118.7
C7—C6—H6C	119.5	C21—C22—H22A	118.7
C11—C7—C8	116.5 (4)	N4—C23—C19	122.2 (4)
C11—C7—C6	119.0 (4)	N4—C23—C24	118.0 (3)
C8—C7—C6	124.6 (4)	C19—C23—C24	119.8 (4)
C9—C8—C7	119.8 (4)	N3—C24—C16	122.9 (4)
C9—C8—H8C	120.1	N3—C24—C23	117.4 (3)
C7—C8—H8C	120.1	C16—C24—C23	119.7 (4)
C8—C9—C10	120.0 (4)	O2—C25—O1	125.9 (4)
C8—C9—H9C	120.0	O2—C25—H25	117.0
C10—C9—H9C	120.0	O1—C25—H25	117.0
N2—C10—C9	121.9 (4)	C25—O1—Cu	108.6 (3)
N2—C10—H10C	119.1	O3—C26—O4	129.0 (5)
C9—C10—H10C	119.1	O3—C26—H26	115.5
N2—C11—C7	123.3 (4)	O4—C26—H26	115.5
N2—C11—C12	116.2 (3)	H5A—O5—H5B	113.5
C7—C11—C12	120.5 (4)	H6A—O6—H6B	102.5
N1—C12—C4	123.4 (3)	H7A—O7—H7B	93.7
N1—C12—C11	117.2 (3)	H8A—O8—H8B	103.3
C4—C12—C11	119.5 (3)	H9A—O9—H9B	94.2
C13—N3—C24	118.3 (3)	H10A—O10—H10B	107.7
C13—N3—Cu	132.4 (3)		

### Hydrogen-bond geometry (Å, °)

**Table:**

*D*—H···*A*	*D*—H	H···*A*	*D*···*A*	*D*—H···*A*
O5—H5A···O8	0.82	2.10	2.874 (5)	160
O5—H5B···O4^i^	0.73	2.10	2.808 (6)	164
O6—H6A···O3	0.74	2.16	2.870 (5)	163
O6—H6B···O10	0.85	2.03	2.810 (5)	153
O7—H7A···O4	0.90	1.95	2.799 (5)	158
O7—H7B···O6^ii^	0.73	2.08	2.794 (6)	165
O8—H8A···O3	0.82	2.12	2.879 (5)	154
O8—H8B···O7^i^	0.76	2.20	2.876 (6)	148
O9—H9A···O2^iii^	0.75	2.05	2.754 (5)	157
O9—H9B···O10^iv^	0.83	2.09	2.827 (6)	148
O10—H10A···O5	0.85	2.03	2.798 (6)	149
O10—H10B···O9	0.82	2.01	2.832 (6)	179

Symmetry codes:  (i) −*x*+1/2, *y*+1/2, −*z*+1/2; (ii) −*x*, −*y*+1, −*z*; (iii) −*x*+1, −*y*+1, −*z*; (iv) −*x*, −*y*+2, −*z*.
